# O-GlcNAc has crosstalk with ADP-ribosylation *via* PARG

**DOI:** 10.1016/j.jbc.2023.105354

**Published:** 2023-10-17

**Authors:** Jie Li, Xiangxiang Liu, Bin Peng, Tingting Feng, Wen Zhou, Li Meng, Shanshan Zhao, Xiyuan Zheng, Chen Wu, Shian Wu, Xing Chen, Xingzhi Xu, Jianwei Sun, Jing Li

**Affiliations:** 1 Beijing Key Laboratory of DNA Damage Response and College of Life Sciences, Capital Normal University, Beijing, China; 2 Center for Life Sciences, Yunnan Key Laboratory of Cell Metabolism and Diseases, School of Life Sciences, State Key Laboratory for Conservation and Utilization of Bio-Resources in Yunnan, Yunnan University, Kunming, Yunnan, China; 3 Guangdong Key Laboratory for Genome Stability & Disease Prevention and Carson International Cancer Center, and Marshall Laboratory of Biomedical Engineering, Shenzhen University School of Medicine, Shenzhen, Guangdong, China; 4 College of Chemistry and Molecular Engineering, Beijing National Laboratory for Molecular Sciences, Peking-Tsinghua Center for Life Sciences, Synthetic and Functional Biomolecules Center, and Key Laboratory of Bioorganic Chemistry and Molecular Engineering of Ministry of Education, Peking University, Beijing, China; 5 State Key Laboratory of Medicinal Chemical Biology, Tianjin Key Laboratory of Protein Sciences, College of Life Sciences, Nankai University, Tianjin, China; 6 College of Life Sciences, Institute of Life Sciences and Green Development, Hebei University, Baoding, Hebei, China

**Keywords:** O-GlcNAcylation, PARG, poly(ADP-ribosyl)ation, hepatocellular carcinoma, c-Myc

## Abstract

O-linked N-acetylglucosamine (O-GlcNAc) glycosylation, a prevalent protein post-translational modification (PTM) that occurs intracellularly, has been shown to crosstalk with phosphorylation and ubiquitination. However, it is unclear whether it interplays with other PTMs. Here we studied its relationship with ADP-ribosylation, which involves decorating target proteins with the ADP-ribose moiety. We discovered that the poly(ADP-ribosyl)ation “eraser”, ADP-ribose glycohydrolase (PARG), is O-GlcNAcylated at Ser26, which is in close proximity to its nuclear localization signal. O-GlcNAcylation of PARG promotes nuclear localization and chromatin association. Upon DNA damage, O-GlcNAcylation augments the recruitment of PARG to DNA damage sites and interacting with proliferating cell nuclear antigen (PCNA). In hepatocellular carcinoma (HCC) cells, PARG O-GlcNAcylation enhances the poly(ADP-ribosyl)ation of DNA damage-binding protein 1 (DDB1) and attenuates its auto-ubiquitination, thereby stabilizing DDB1 and allowing it to degrade its downstream targets, such as c-Myc. We further demonstrated that PARG-S26A, the O-GlcNAc-deficient mutant, promoted HCC in mouse xenograft models. Our findings thus reveal that PARG O-GlcNAcylation inhibits HCC, and we propose that O-GlcNAc glycosylation may crosstalk with many other PTMs.

O-linked N-acetylglucosamine (O-GlcNAc) glycosylation is a post-translational modification (PTM) that is installed onto the Ser/Thr residues of nucleocytoplasmic proteins to mediate protein-protein interactions, and change protein enzymatic activity, stability or localization (1, 2). Since its discovery almost 4 decades ago, investigators have identified about 5000 substrates, through which O-GlcNAc has crosstalk with phosphorylation and ubiquitination in myriad biological processes (1, 2). O-GlcNAcylation has a sole writer, O-GlcNAc transferase (OGT), and the only eraser is O-GlcNAcase (OGA). This duo regulates many aspects of DNA metabolism, especially cell cycle and DNA damage response (3, 4).

Here we attempted to address whether O-GlcNAc has crosstalk with other PTMs. We reasoned that among the 300 known modifications (5), other forms of PTM might interact with O-GlcNAc. We focused on ADP-ribosylation, whose donor group is nicotinamide adenine dinucleotide (NAD^+^) (6). Writers, such as poly(ADP-ribose) polymerases (with PARP1 being the founding member), transfer the ADP-ribose moiety from NAD^+^ to protein substrates, resulting in protein mono-ADP-ribosylation (MARylation) or poly-ADP-ribosylation (PARylation) (6). Its erasers include poly-ADP-ribose glycohydrolase (PARG) and ADP-ribosyl-acceptor hydrolase 3 (ARH3), with the former degrading the PAR chains and the latter hydrolyzing MARylation (7).

Both O-GlcNAcylation and PARylation function in chromatin metabolism and stress response, which are critical for DNA repair, cell cycle, and cell death. Both PTMs are enriched in the nucleus and chromatin, with fundamental roles in the DNA damage response (4, 8). In respect to their localization patterns, OGT, OGA, PARP1, and PARG are all recruited to DNA damage sites (9, 10). In terms of nutrient status, O-GlcNAc has been deemed as a rheostat for glucose, amino acid, glutamine, fatty acid, and nucleotide metabolism, and PARG-mediated de-PARylation has been proposed essential to release ATP to provide energy for local DNA repair events (11).

PARP inhibitors are intensely investigated and four have been widely used in clinical settings (12). Synthetic lethality has been observed between PARP inhibitors and DNA repair protein mutations (6), and thus has been exploited for treating tumors and other non-cancerous diseases (12). Besides PARP inhibitors, PARG inhibitors have also been widely studied for clinical purposes (13), which target replication stress in tumors (14). By suppressing replication fork progression in cancer cells, new PARG inhibitors are developed to sensitize tumors to radiation-induced damage and thus cell death (15). Besides proteins, both DNA (16) and RNA (17) are also subject to ADP-ribosylation (18), suggesting that PARP inhibitors (and maybe PARG inhibitors) can be further studied to assess their effects on DNA and RNA.

In this report, we focused on PARG, the sole eraser for PARylation. PARG is nuclear due to a nuclear localization signal (NLS) at its N-terminus (6). During DNA damage, PARG is recruited through both PAR chains and proliferating cell nuclear antigen (PCNA) to DNA damage sites (10, 19), where it executes dePARylation activities (20). In a recent report where immunotherapy was used for patients with advanced hepatocellular carcinoma (HCC), PARG inhibition was shown to have a synergistic effect with anti-programmed cell death 1 (PD1) therapy (21). Its underlying mechanism is that PARG dePARylates DNA damage-binding protein 1 (DDB1) in hepatocytes, which promotes DDB1 auto-ubiquitination and stabilizes its downstream targets (*e.g.*, c-Myc) (21). Both mRNA and protein levels of PARG are upregulated in patients with HCC, which correlate with HCC prognosis (21), suggesting that PARG functions as an oncogene in HCC.

Herein we found that PARG is O-GlcNAcylated. Through electron-transfer dissociation (ETD) mass spectrometry (MS), we identified Ser-26 as a major O-GlcNAcylation site. PARG O-GlcNAcylation promotes its nuclear retention and chromatin recruitment during DNA damage. In HCC cells, PARG O-GlcNAcylation promotes DDB1 PARylation, downregulates its auto-ubiquitination and increases its stability, resulting in decreased c-Myc. Our work suggests that PARG O-GlcNAcylation suppresses HCC, revealing a link between O-GlcNAcylation and PARylation.

## Results

### PARG interacts with OGT and is O-GlcNAcylated

**Figure 1:**
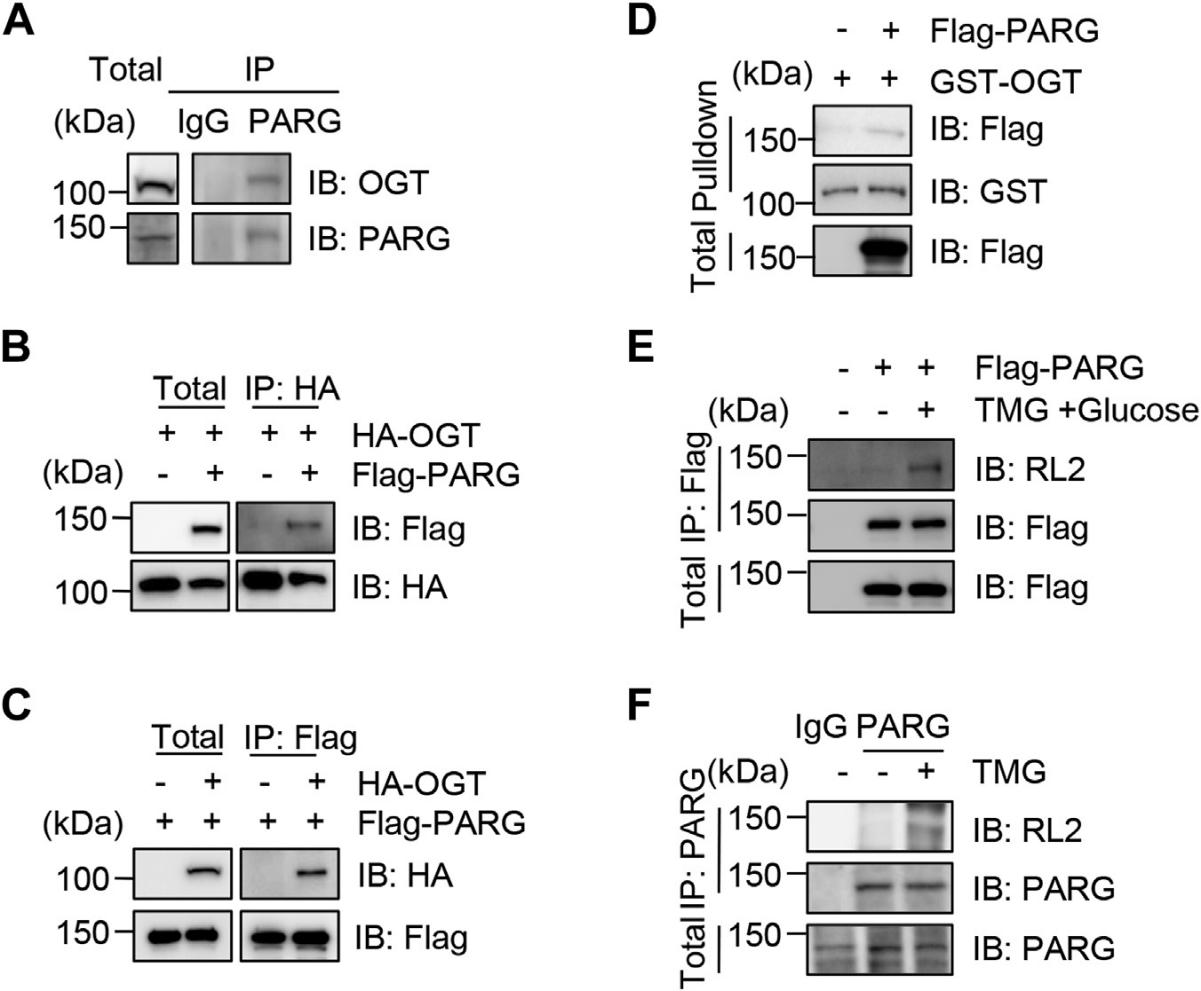
**PARG interacts with OGT**. *A*, endogenous PARG and OGT co-immunoprecipitate. 293T cell lysates were immunoprecipitated with anti-PARG antibodies and immunoblotted with anti-OGT and anti-PARG antibodies. *B* and *C*, exogenous HA-OGT and Flag-PARG co-immunoprecipitate reciprocally. Cells were transfected with HA-OGT and Flag-PARG plasmids, and the lysates were subject to immunoprecipitation and immunoblotting with the antibodies indicated. *D*, cells were transfected with Flag-PARG plasmids, and the cell lysates were incubated with recombinant GST-OGT proteins. *E*, cells were transfected with Flag-PARG and treated with the OGA inhibitor Thiamet-G (TMG) plus glucose as previously described (22). Then, the anti-Flag immunoprecipitates were immunoblotted with an anti-O-GlcNAc antibody, RL2. *F*, endogenous PARG is O-GlcNAcylated. Cells were treated with TMG, and the lysates were immunoprecipitated with anti-PARG antibodies. All western blots were repeated at least three times.

To examine if PARG is O-GlcNAcylated, we first assessed if PARG associates with OGT. 293T cell lysates were immunoprecipitated (IPed) with anti-PARG antibodies and immunoblotted (IBed) with anti-OGT antibodies. As Figure 1*A* revealed, endogenous PARG interacts with OGT. Then cells were transfected with HA-OGT and Flag-PARG plasmids, and the cell lysates were IPed with anti-HA (Fig. 1*B*) or anti-Flag (Fig. 1*C*) antibodies; the results showed a reciprocal coIP between the two overexpressed proteins. Recombinant GST-OGT proteins were also utilized in GST-pulldown assays (Fig. 1*D*), and again GST-OGT could pulldown Flag-PARG, indicative of the binding between OGT and PARG. Then we directly tested if PARG is O-GlcNAcylated (Fig. 1*E*). By supplementing the medium with glucose and Thiamet-G (TMG, OGA inhibitor) as previously described (22), we observed a crisp RL2 (a pan-O-GlcNAc antibody) band, suggesting that PARG is O-GlcNAcylated. We also examined the glycosylation of endogenous PARG (Fig. 1*F*). When cells were treated with TMG, endogenous PARG was also O-GlcNAcylated (Fig. 1*F*). Taken together, PARG is O-GlcNAcylated.

### PARG is O-GlcNAcylated at Ser-26 as identified by ETD MS

**Figure 2:**
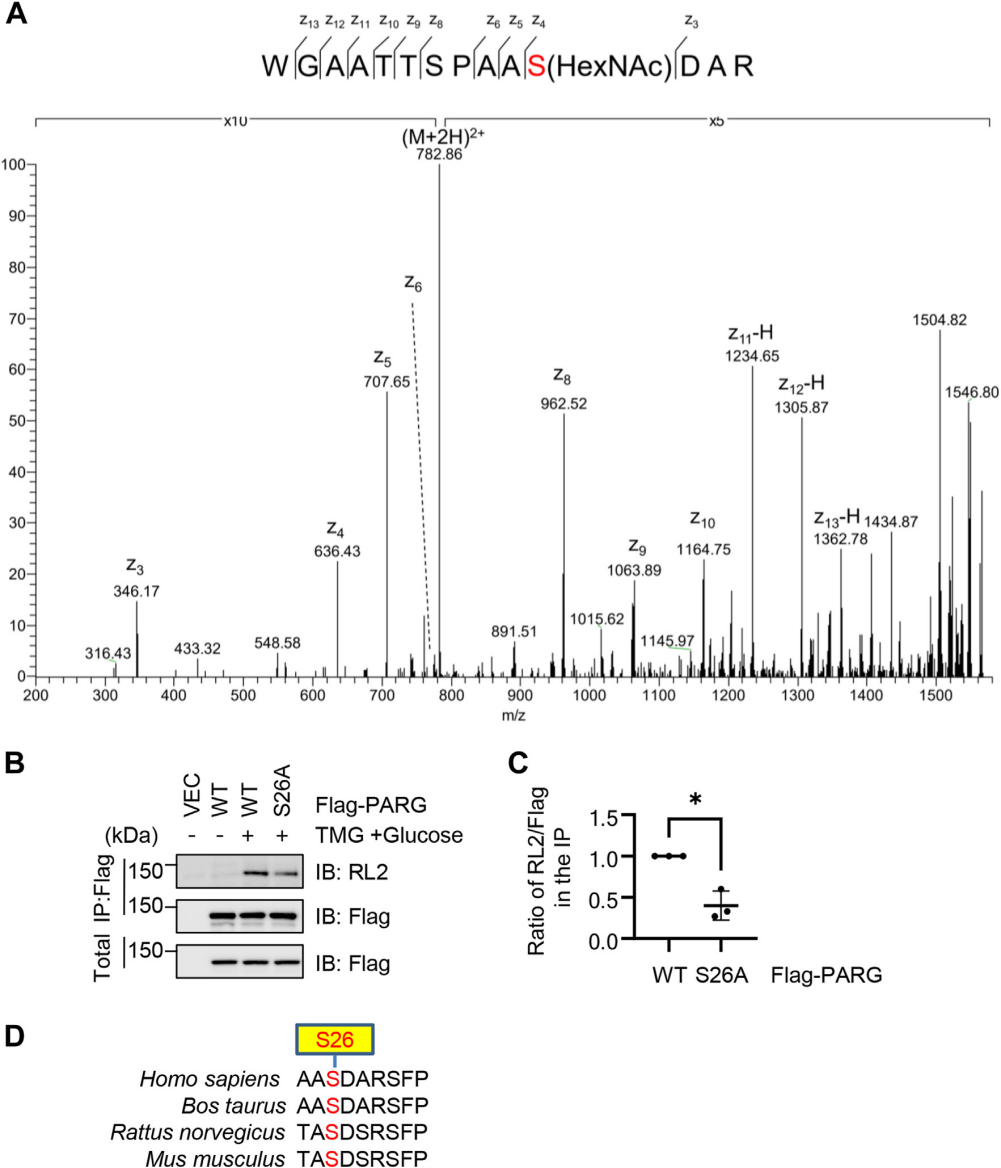
**PARG is O-GlcNAcylated at Ser26**. *A*, electron transfer dissociation (ETD) Mass Spectrometry revealed that Ser26 of PARG could be an O-GlcNAcylation sites. *B* and *C*, 293T cells were transfected with Flag-PARG-WT or -S26A plasmids and the lysates were subject to immunoprecipitation and immunoblotting assays as indicated. Quantitation was carried out with a *t* test; ∗ indicates *p* < 0.05. *D*, conservation of Ser26 in different species.

To identify the O-GlcNAc site on PARG, Flag-PARG was transfected into 293T cells, and the medium was again enriched for O-GlcNAcylation by TMG plus glucose treatment (22). The cell lysates were subject to immunoprecipitation with anti-Flag antibodies, and the immunoprecipitated Flag-PARG was analyzed by ETD MS. The results revealed an O-GlcNAc peptide where Ser-26 is the glyco-site (Fig. 2*A*), close to its NLS. We constructed the S26A mutant accordingly, and the RL2 Western blotting showed ∼50% reduction in the mutant (Fig. 2, *B* and *C*), suggesting that Ser-26 is a major O-GlcNAc site on PARG; it is possible that PARG harbors other O-GlcNAc sites. Interestingly, Ser-26 is conserved in some mammals (Fig. 2*D*), but not in the fly or other model organisms.

### *Drosophila* PARG (dPARG) is O-GlcNAcylated

We also attempted to examine PARG O-GlcNAcylation in *Drosophila.* Previous investigations have shown that *PARG* knockout mice show embryonic lethality (23), and *PARG* disruption also results in lethal phenotypes in *Drosophila melanogaster* at 25 °C (24), suggesting that PARG may be functionally conserved and essential during animal development. We compared the sequence of human PARG and *Drosophila* PARG (dPARG) and found them to be relatively conserved (Fig. S1). We also analyzed dPARG O-GlcNAcylation by ETD MS and identified seven sites (Table S1) (Fig. S2), which are also close to the predicted NLS of dPARG (25). As MS cannot tell apart the S651/S652/T653 or T653/T654/S655 sites (Fig. S2), we decided to generate a S649A/S651A/S652A/T653A/T654A/S655A (6A) mutant of dPARG, as it is a common practice in the field and was applied to the study of estrogen receptor beta (26). We found that dPARG-6A significantly attenuated O-GlcNAcylation levels (data not shown). To explore the effect of O-GlcNAc on dPARG function, we generated a genome deletion allele of d*Parg* (d*Parg*^*del*^) by CRISPR-CAS9. Similar to the recorded allele d*Parg*^*27.1*^ (24), d*Parg*^*del*^ is also lethal at 25°C. d*Parg*^*del*^ is rescued by expressing either wild-type (WT) human PARG or WT dParg, demonstrating that PARG is functionally conserved from insects to humans. Unfortunately, the human O-GlcNAc-defective PARG-S26A rescues the lethality of *Parg*^*del*^ as WT PARG, indicating that O-GlcNAc of PARG might not be essential for normal fly development. We reason that O-GlcNAcylation of dPARG probably functions under certain conditions such as nutrition stress and/or pathogen invasion. The role of dPARG O-GlcNAcylation still warrants further investigation.

### O-GlcNAcylation promotes nuclear retention and chromatin association of PARG

**Figure 3:**
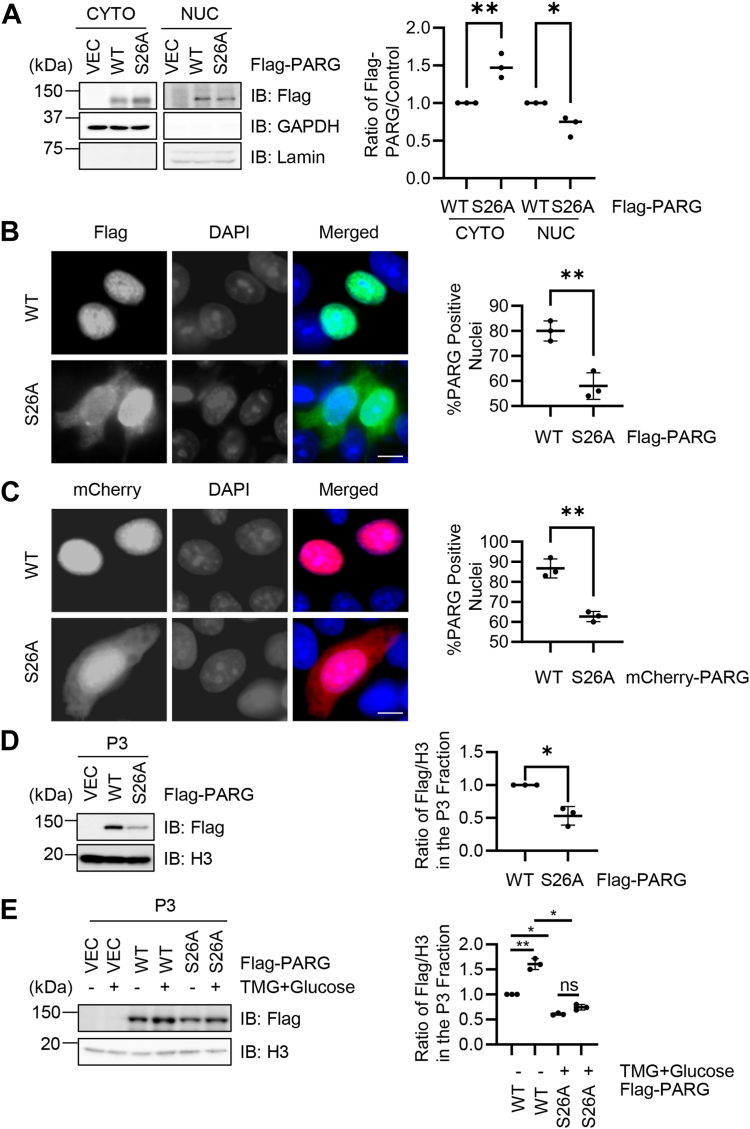
**O-GlcNAcylation promotes nuclear retention of PARG**. *A*, 293T cells were transfected with Flag-PARG-WT or -S26A, and nuclear/cytoplasmic fractionation assays were carried out. *B*, immunofluorescence microscopy staining shows the nuclear localization of PARG-WT, whereas a portion of Flag-PARG-S26A localizes to the cytosol. Scale bar, 10 μM. *C*, microscopic images of mCherry-PARG-WT or -S26A, showing that more S26A localizes to the cytoplasm. Immunofluorescence experiments were repeated three times, with 100 cells per experiment. Scale bar, 10 μM. *D*, cells were transfected with Flag-PARG-WT or -S26A, and the chromatin-bound P3 fraction was extracted. *E*, cells were transfected with Flag-PARG-WT or -S26A, and treated with TMG plus glucose or not treated. Then the P3 fraction was extracted. Quantitation in (*A*) and (*E*) was done with a two-way ANOVA, in (*B*–*D*) was done with Student’s *t* test. ∗ indicates *p* < 0.05; ∗∗indicates *p* < 0.005. All western blots were repeated at least three times.

O-GlcNAcylation has been linked with the nuclear shuttling of many proteins (27, 28), so we examined whether the same holds true for PARG O-GlcNAcylation. Nuclear cytoplasmic fractionation was carried out and the O-GlcNAc-deficient S26A mutant manifested a decreased nuclear portion with an increased cytoplasmic fraction (Fig. 3*A*). Then we used immunofluorescence microscopy to examine PARG localization (Fig. 3*B*). WT PARG localizes to the nucleus, consistent with its role in degrading PARylation in the nucleus (20). In stark contrast, PARG-S26A dramatically increased its cytoplasmic localization (Fig. 3*B*), indicating that O-GlcNAcylation promotes nuclear localization of PARG. We also constructed mCherry-PARG-WT and -S26A. When examined under a microscope, mCherry-PARG-S26A also showed more localization in the cytosol (Fig. 3*C*).

As PARG has an important role in DNA damage (6, 29), we further examined chromatin binding of PARG. Chromatin fractionation was performed and PARG was assessed in the chromatin-bound P3 fraction. The PARG-S26A mutant significantly decreased chromatin binding (Fig. 3*D*). We also utilized the TMG + Glucose method to enrich for O-GlcNAcylation and found that chromatin-bound PARG was elevated, but the O-GlcNAc-deficient S26A mutant was not (Fig. 3*E*). These findings suggest that O-GlcNAcylation promotes chromatin association of PARG.

### O-GlcNAcylation enhances PARG chromatin binding upon DNA damage

**Figure 4:**
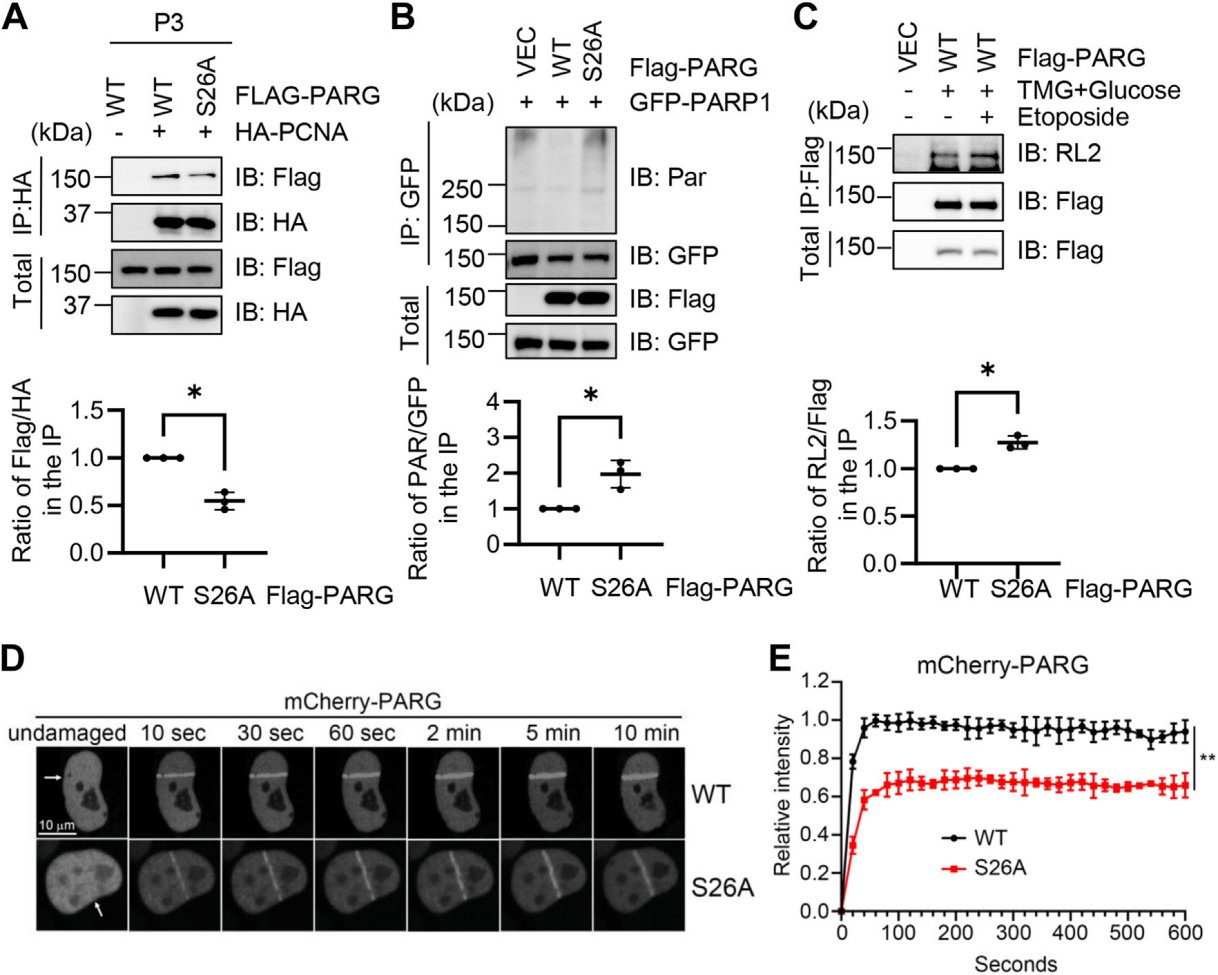
**O-GlcNAcylation enhances recruitment of PARG to DNA damage sites.***A*, 293T cells were transfected with Flag-PARG-WT or -S26A, together with HA-PCNA. *B*, cells were transfected with Flag-PARG and GFP-PARP1. *C*, 293T cells were transfected with Flag-PARG-WT, treated with TMG plus glucose and Etoposide (10 μM, 2 h). *D*–*E*, U2OS cells were transfected with mCherry-PARG, treated with laser micro-irradiation (*arrows*), and the mCherry signals quantitated. Scale bar, 10 μM. The laser microirradiation experiments were repeated three times, with 10 cells per experiment. Quantitation in (*A*–*C*) was done with Student’s *t* test, in *E* was done with a two-way ANOVA. ∗ indicates *p* < 0.05; ∗∗indicates *p* < 0.005. All western blots were repeated at least three times.

The results in Figure 3, *C* and *D* prompted us to further examine the role of PARG O-GlcNAcylation in DNA damage. As PARG is recruited to DNA damage sites by PCNA (10, 19), we tested the interaction between PARG and PCNA in the chromatin-bound fraction (Fig. 4*A*). The results showed that S26A downregulated binding with PCNA. PARG catalyzes the dePARylation reaction, so we used the PARP1 protein as the substrate to measure the effect of PARG O-GlcNAcylation. As shown in Figure 4*B*, PARG-WT efficiently hydrolyzed the PAR chain on PARP1, but the PARG-S26A mutant did not.

We wondered whether PARG O-GlcNAcylation increases during DNA damage, so we used Etoposide to treat the cells and induce DNA damage (Fig. 4*C*). Consistently, Etoposide increased PARG O-GlcNAcylation by about 20% (Fig. 4*C*). Then we directly measured the recruitment of PARG to DNA damage sites *via* laser micro-irradiation. mCherry-PARG was used to visualize PARG recruitment. Upon laser micro-irradiation, PARG was accumulated at DNA damage stripes within 10 s, while the intensity of PARG-S26A was decreased to ∼60% (Fig. 4, *D* and *E*). Taken together, our analysis revealed that O-GlcNAcylation promotes chromatin recruitment of PARG to DNA damage sites, probably *via* PCNA.

### O-GlcNAcylation upregulates DDB1 PARylation and destabilizes c-Myc in HCC cells

**Figure 5:**
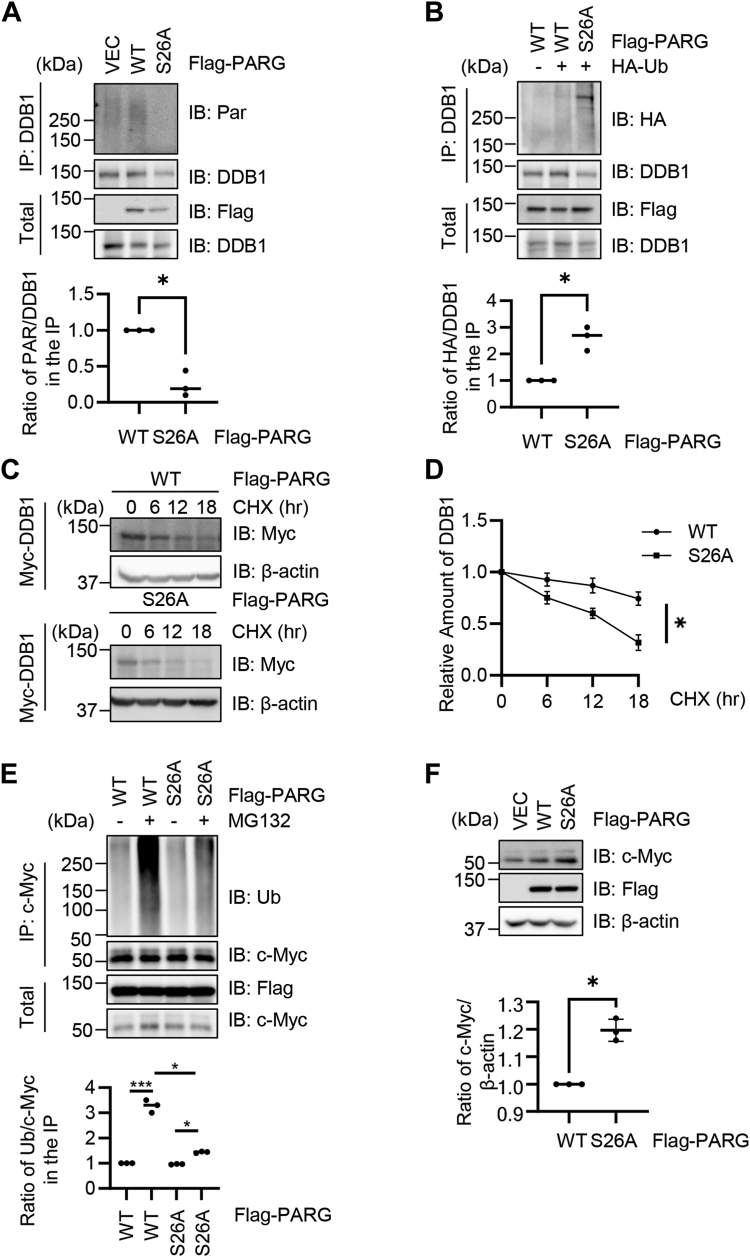
**PARG O-GlcNAcylation attenuates c-Myc levels by maintaining DDB1 stability in HCC Huh7 cells**. *A*, Huh7 cells were transfected with Flag-PARG-WT and -S26A plasmids. The lysates were immunoprecipitated with anti-DDB1 antibodies and immunoblotted with anti-Par antibodies. *B*, Huh7 cells were transfected with Flag-PARG-WT and -S26A plasmids, together with HA-Ub. Then the anti-DDB1 immunoprecipitates were immunoblotted with anti-HA antibodies to detect the ubiquitination levels. *C* and *D*, Huh7 cells were transfected with Myc-DDB1 plasmids together with Flag-PARG-WT or -S26A plasmids, and then cycloheximide (CHX) was added to block new protein synthesis. *E*, Huh7 cells were transfected with Flag-PARG-WT and -S26A plasmids, and the proteasome inhibitor MG132 was added to block ubiquitin-mediated protein degradation. The anti-c-Myc immunoprecipitates were immunoblotted with anti-Ub antibodies. *F*, Huh7 cells were transfected with Flag-PARG-WT and -S26A plasmids, and c-Myc levels were detected and quantitated. Quantitation in (*A*, *B*, and *F*) was done with Student’s *t* test, in (*D*) and (*E*) was done with a two-way ANOVA. ∗ indicates *p* < 0.05. All Western blots were repeated at least three times.

As recently DDB1 has been shown to be a PARG substrate in HCC (21), we used HCC Huh-7 cell lines and examined the potential effects of PARG O-GlcNAcylation on DDB1. We reasoned that as PARG-S26A increases cytosolic localization, it would further hydrolyze the PAR chain on DDB1. Indeed, DDB1 PARylation was almost abolished in PARG-S26A transfected cells (Fig. 5*A*). As DDB1 PARylation counteracts its auto-ubiquitination (21), we then assessed DDB1 ubiquitination and its stability (Fig. 5, *B*–*D*). In PARG-S26A transfected cells, DDB1 ubiquitination was upregulated (Fig. 5*B*), resulting in decreased protein stability as measured by cycloheximide (CHX) pulse-chase assays (Fig. 5, *C* and *D*).

A downstream target of the DDB1-CUL4 E3 ligase is c-Myc (21). And PARG-S26A transfection significantly attenuated c-Myc ubiquitination (Fig. 5*E*), leading to increased c-Myc protein levels (Fig. 5*F*). In sum, we found that increased cytosolic PARG in HCC cells decreased DDB1 PARylation and increased DDB1 auto-ubiquitination. Destabilized DDB1 attenuated c-Myc ubiquitination and elevated c-Myc levels.

### PARG O-GlcNAcylation inhibits HCC *in vivo*

**Figure 6:**
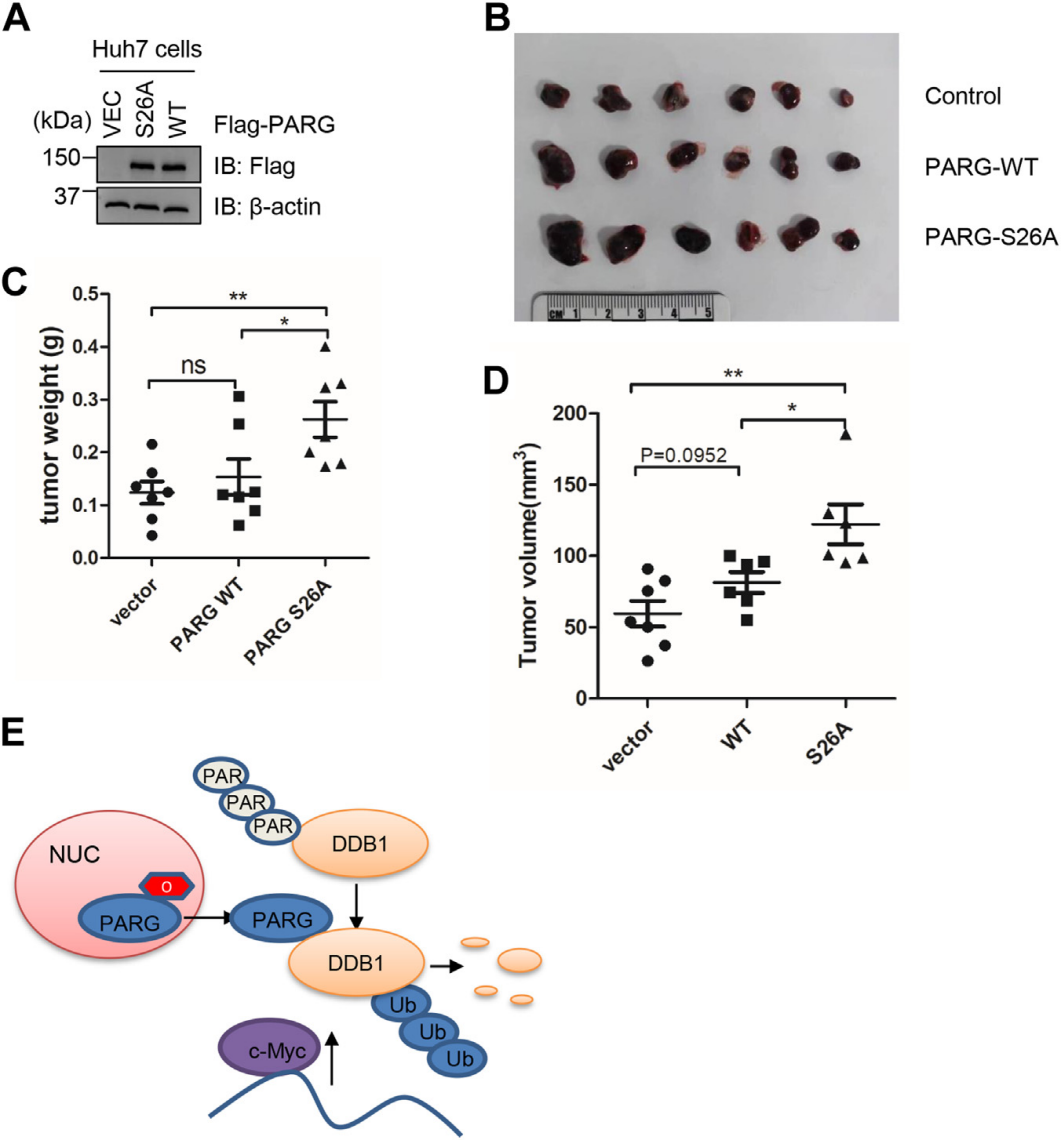
**The PARG-S26A mutant increases HCC in mouse xenograft models**. *A*, Huh7 cells stably expressing PARG-WT and -S26A plasmids were constructed. *B*–*D*, Xenografts in nude mice. PARG-WT and -S26A Huh7 cells were injected into nude mice, and the tumors generated were photographed. Tumor weights were quantitated in (*C*), and tumor sizes were quantitated in (*D*). Quantitation was done with a one-way Anova. ∗*p* < 0.05; ∗∗*p* < 0.01. *E*, model depicting the role of PARG O-GlcNAcylation. O-GlcNAcylation promotes PARG nuclear retention. The O-GlcNAc-deficient PARG localizes to the cytoplasm, where it dePARylates DDB1 in HCC cells, increases DDB1 ubiquitination, and attenuates its stability. DDB1 substrates, such as c-Myc, are thus upregulated, leading to HCC.

We wondered whether the OGT-PARG-DDB1-c-Myc axis functions *in vivo* and thus constructed HCC cells stably expressing PARG-WT and -S26A plasmids (Fig. 6*A*). These cells were injected into nude mice to perform xenograft assays (Fig. 6*B*). PARG-S26A significantly increased the tumor size and weight compared to WT (Fig. 6, *C* and *D*), suggesting that upregulated c-Myc in S26A cells promotes HCC (Fig. 6*E*).

## Discussion

In this report, we examined whether O-GlcNAcylation interacts with PARylation, and revealed that PARG is O-GlcNAcylated. The glycosylation modification ensures the nuclear localization of PARG and is essential for its recruitment to DNA damage sites. Increased cytoplasmic PARG-S26A almost abolishes PARylation of DDB1 in HCC, thus DDB1 auto-ubiquitination is elevated. Hence, downregulated DDB1 stabilizes its substrate (*e.g.* c-Myc), resulting in HCC tumorigenesis (Fig. 6*E*).

The relationship between PARylation and ubiquitination is context-dependent. PARylation of DDB1 counteracts auto-ubiquitination in HCC cells (21), but in the case of p21, PARylation by tankyrase actually promotes ubiquitination and subsequent proteasome-mediated degradation (30). During DNA damage, topoisomerase I (TOP1) DNA-protein crosslink (TOP1-DPC) is also PARylated, which recruits Ubiquitin-specific protease 7 (USP7) to de-ubiquitinate TOP1-DPC (31). We think that PARylation may affect ubiquitination by altering protein-protein interaction, thus exerting distinct effects on different substrates.

The same holds true for O-GlcNAcylation. Recently, about 75 proteins were identified to be hyper-stably O-GlcNAcylated in a chemoproteomic study and O-GlcNAcylation was shown to promote their stability (32). However, in our recent report, Polo-like kinase 1 (PLK1) was found to be O-GlcNAcylated, which enhances its degradation (33), consistent with previous reports (34). As PTMs generally alter protein localization, stability, or protein–protein interaction, more aspects of O-GlcNAcylation may be explored to reveal its crosstalk with PARylation or other PTMs.

For therapeutic purposes, PARG inhibitors could be exploited for HCC suppression and they could be used synergistically with anti-PD-1 antibodies (21). DDB1 was recently shown to be PARylated upon DNA damage, which is dependent on double-strand breaks (DSBs) (35). Further, PARG inhibition causes DSB repair defects in HCC cells (35). We found that PARG-S26A decreased recruitment to DSB sites (Fig. 4, *C* and *D*), suggesting that O-GlcNAcylation functions positively for PARG-mediated DNA damage repair. In sum, our findings reveal a new layer of regulation of PARG, which could be utilized for targeting PARG to treat HCC.

## Experimental procedures

### Cell culture, antibodies, and plasmids

U2OS and 293T cells were purchased from ATCC. The cell lines were validated using STR profiling and free from *mycoplasma* contamination for all experiments. *PARP1* (20) and *PARG* (36) plasmids were previously described. Antibodies were as follows: RL2 (Abcam, AB2739), anti-c-Myc (ProteinTech, 10828-1-AP), anti-PARG (Santa Cruz, Sc-398563), anti-DDB1 (GeneTex, GTX100130), anti-PAR (Trevigen, 4335-MC-100), anti-ubiquitin (PTM Biolabs, #PTM-1106RM), anti-Flag (Sigma, F1084), anti-GST (Gene Script, A00865), anti-HA (Bethyl Laboratories, A190–108A), anti-OGT (Abcam, ab96718), and anti-β-actin (Sigma, A5441). PARG mutant plasmids were generated using specific primers (sequences available upon request) following the manufacturer's instructions (QuickChange II, Stratagene).

### Immunoprecipitation and immunoblotting assays

Immunoprecipitation and immunoblotting experiments were performed as described before (37). The following primary antibodies were used for immunoblotting: anti-HA (1:1000), anti-FLAG M2 (Sigma) (1:1000), anti-DDB1(1:1000), anti-Par (1:1000), anti-c-Myc (1:3000), anti-OGT (1:1000), anti-PARG (1:5000), and anti-Ub (1:1000). Peroxidase-conjugated secondary antibodies were from JacksonImmuno Research. The ECL detection system (Amersham) was used for immunoblotting. LAS-4000 was employed to detect signals and quantitated using Multi Gauge software (Fujifilm). All Western blots were repeated at least three times.

### LC-MS/MS analysis

#### Sample preparation

The gel band pieces were dehydrated in acetonitrile, incubated in 10 mM DTT in 50 mM ammonium bicarbonate at 56 °C for 40 min, incubated in 55 mM iodoacetamide in 50 mM ammonium bicarbonate at ambient temperature for 1 h in the dark, and finally dehydrated again. Then the gel pieces were digested in-gel with 2 ng/μl sequencing grade trypsin in 50 mM ammonium bicarbonate overnight at 37 °C. The resulting peptides were extracted twice with 5% formic acid/50% acetonitrile, then vacuum-centrifuged to dryness. All samples were resuspended in 0.1% FA in water prior to LC-MS/MS analysis.

#### LC-MS/MS parameters

Peptides were separated using a loading column (100 μm × 2 cm) and a C18 separating capillary column (75 μm × 15 cm) packed in-house with Luna 3 μm C18 (2) bulk packing material (Phenomenex). The mobile phases (A: water with 0.1% formic acid and B: 100% acetonitrile with 0.1% formic acid) were driven and controlled by an EASY-nLC 1000 system (Thermo Fisher Scientific). The LC gradient was held at 2% for 1 min of the analysis, followed by an increase from 2% to 7% B from 1 to 2 min, an increase from 7% to 35% B from 2 to 62 min, and an increase from 35% to 75% B from 62 to 66 min.

MS data were acquired in data-dependent mode with a full MS scan (300–1700 M/z) in FT mode at a resolution of 60,000 followed by ETD MS/MS scans on the 10 most abundant ions with multiple charges in the initial MS scan. Automatic gain control (AGC) targets were 1e6 ions for Orbitrap scans and 5e4 for MS/MS scans. For dynamic exclusion, the following parameters were used: isolation window, 2 m/z; repeat count, one; repeat duration, 25 s; and exclusion duration, 25 s. The ETD activation time was 150 ms. Charge state dependent time and supplemental activation for ETD were enabled.

#### Data analysis

Data processing was carried out using Thermo Proteome Discoverer 2.4 using a SwissProt *Homo sapiens* database (https://www.expasy.org/) (TaxID = 9606 and subtaxonomy, 42,253 protein sequences). Carbamidomethyl (Cys) was chosen as a static modification, and oxidation (Met) was chosen as a variable modification. Mass tolerance was 10 ppm for precursor ions and 0.6 Da for fragment ions. Maximum missed cleavages were set as 2. Peptide spectral matches (PSM) were validated using the Percolator algorithm, based on q-values at a 1% FDR at both the peptide and protein levels.

### Indirect immunofluorescence staining

Indirect immunofluorescence staining was carried out as described previously (38). Antibody dilutions were 1:1000 for mouse anti-Flag. The nuclei were stained with DAPI. All immunofluorescence experiments were repeated three times, with 100 cells per experiment.

### Laser microirradiation

U2OS cells were grown on a confocal dish and then irradiated with a 365 nm pulsed nitrogen UV laser (16 Hz pulse, 50% laser output) (Micropoint, Andor). Real-time images were taken every 10 s with a Dragonfly confocal imaging system (Andor). Images were quantitated with ImageJ (ImageJ, RRID:SCR_003070) (link). The laser microirradiation experiments were repeated three times, with 10 cells per experiment.

### Mouse xenograft analysis

A series of pilot studies was first undertaken to test the feasibility of transplanting Huh7 cell lines. Huh7 cells were infected with lentivirus of pHAGE-FLAG-VET, pHAGE-FLAG-PARG and pHAGE-FLAG-S26A, respectively, and then screened by puromycin (10 mg/ml) to obtain stable Huh7 cells for VEC, PARG-WT and PARG-S26A. The transplantation protocol followed published guidelines (39), and 6-week-old nude mice were given a single injection in both flanks of 1.2 × 10^6^ cells, which were in the log phase of growth and resuspended in Matrigel (GLPBIO). After inoculation, tumor volumes were measured from day 5 to day 14. On the 14th day, tumors were dissected, and the tumor volume was calculated according to the following formula: volume = ((4 × 3.14/3) × (L/2) × (W/2) × (D))/2. The tumor weights were measured at necropsy. The mice were obtained from the Animal Research and Resource Center, Yunnan University, with the Certification NO. SCXK(Dian) K2021 to 0001. All animal work procedures were approved by the Animal Care Committee of Yunnan University.

## Data availability

The mass spectrometry proteomics data have been deposited to the ProteomeXchange Consortium *via* the PRIDE (40) partner repository with the dataset identifier PXD041119 and 10.6019/PXD041119.

## Supporting information

This article contains supporting information.

## Conflicts of interest

The authors declare that they have no conflicts of interest with the contents of this article.
