# Supplementary material for: O-GlcNAc has crosstalk with ADP-ribosylation via PARG
Source: J Biol Chem. 2023 Oct 17;299(11):105354. doi: 10.1016/j.jbc.2023.105354 (PMC10654028; doi:10.1016/j.jbc.2023.105354)
Supplement: Supplemental figure 1 [file mmc1.pptx]

## Slide 1
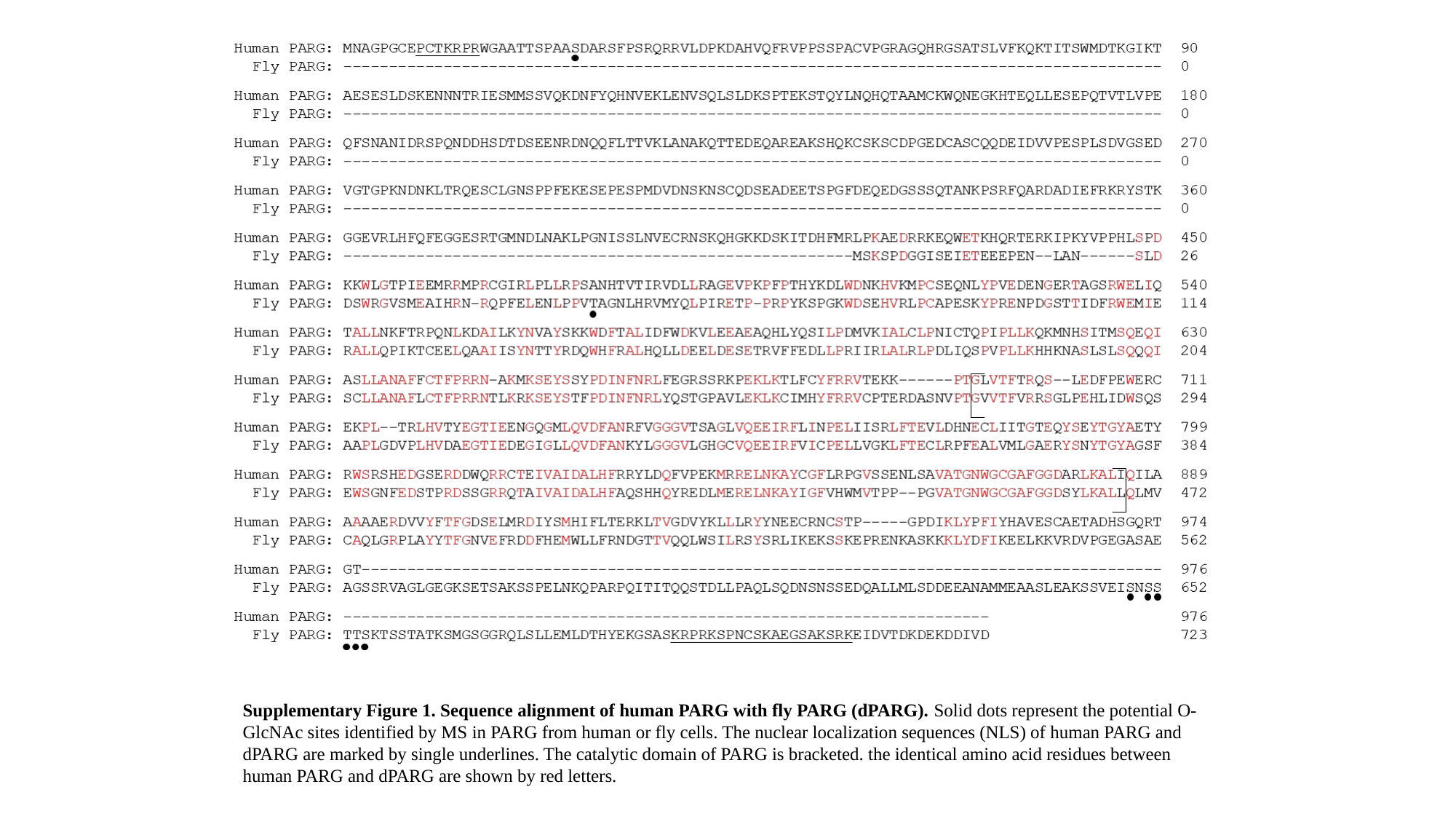

Supplementary Figure 1. Sequence alignment of human PARG with fly PARG (dPARG). Solid dots represent the potential O-GlcNAc sites identified by MS in PARG from human or fly cells. The nuclear localization sequences (NLS) of human PARG and dPARG are marked by single underlines. The catalytic domain of PARG is bracketed. the identical amino acid residues between human PARG and dPARG are shown by red letters.
