# Supplementary material for: O-GlcNAc has crosstalk with ADP-ribosylation via PARG
Source: J Biol Chem. 2023 Oct 17;299(11):105354. doi: 10.1016/j.jbc.2023.105354 (PMC10654028; doi:10.1016/j.jbc.2023.105354)
Supplement: Supplemental figure 2 [file mmc2.pptx]

## Slide 1
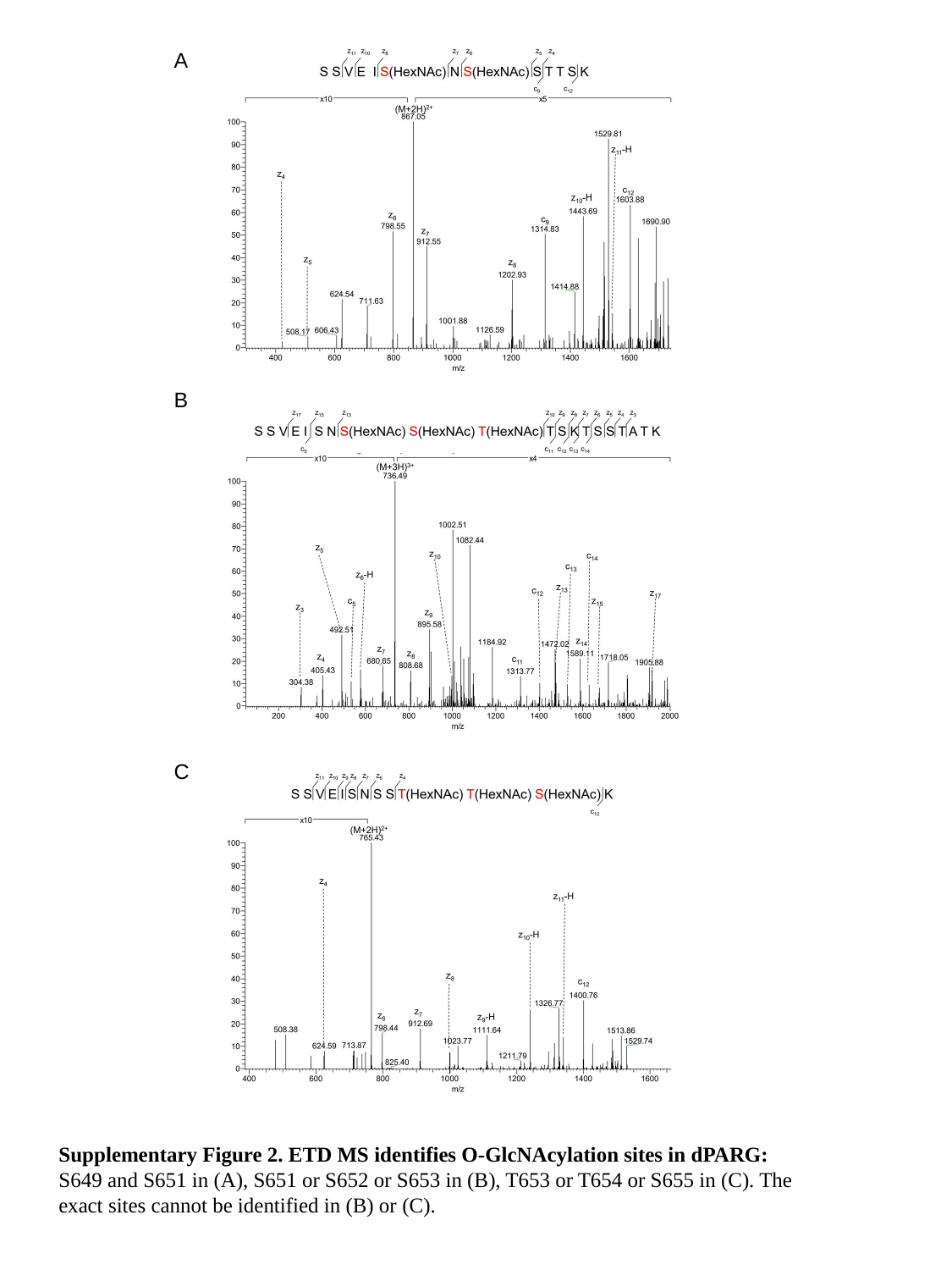

A
B
C
Supplementary Figure 2. ETD MS identifies O-GlcNAcylation sites in dPARG: S649 and S651 in (A), S651 or S652 or S653 in (B), T653 or T654 or S655 in (C). The exact sites cannot be identified in (B) or (C).
